# Design and analysis of Maxwell fisheye lens based beamformer

**DOI:** 10.1038/s41598-021-02058-9

**Published:** 2021-11-23

**Authors:** Muhammad Ali Babar Abbasi, Rafay I. Ansari, Gabriel G. Machado, Vincent F. Fusco

**Affiliations:** 1 Institute of Electronics, Communications and Information Technology (ECIT), Queen’s University Belfast, Belfast, UK; 2 Department of Computer and Information Sciences, Northumbria University, Newcastle upon Tyne, NE1 8ST UK

**Keywords:** Electrical and electronic engineering, Applied physics

## Abstract

Antenna arrays and multi-antenna systems are essential in beyond 5G wireless networks for providing wireless connectivity, especially in the context of Internet-of-Everything. To facilitate this requirement, beamforming technology is emerging as a key enabling solution for adaptive on-demand wireless coverage. Despite digital beamforming being the primary choice for adaptive wireless coverage, a set of applications rely on pure analogue beamforming approaches, e.g., in point-to-multi point and physical-layer secure communication links. In this work, we present a novel scalable analogue beamforming hardware architecture that is capable of adaptive 2.5-dimensional beam steering and beam shaping to fulfil the coverage requirements. Beamformer hardware comprises of a finite size Maxwell fisheye lens used as a scalable feed network solution for a semi-circular array of monopole antennas. This unique hardware architecture enables a flexibility of using 2 to 8 antenna elements. Beamformer development stages are presented while experimental beam steering and beam shaping results show good agreement with the estimated performance.

## Introduction

The vision for Beyond 5G (B5G) networks paves the path towards ultra-reliable low-latency (URLLC) communications. Recent times have seen a sharp rise in the number of connected devices, especially in the context of internet-of-everything (IoE)^1^. Wireless communication technologies have found widespread use in different applications that impact agriculture, transport and healthcare to name a few. Therefore, new and innovative techniques have been proposed for providing seamless coverage and improved link quality to provide high data rates. In the new communication infrastructure, multi antenna systems have been utilized to provide better antenna gain, enabling enhanced communication link quality^2^. Additionally, providing seamless connectivity becomes more challenging for applications in mobile platforms, e.g. unmanned aerial vehicle (UAV), vehicular and satellite networks where electrically scanned arrays are beneficial. To overcome these challenges, beamforming techniques have been utilized to enhance the network coverage and overcome the limitations due to interference and path loss. Similarly, beam steering allows the transmissions to be directed at a particular angle(s), ensuring seamless connectivity for mobile devices^3^.

Three well-known beamforming techniques are analog, digital and hybrid beamforming. Generally a single radio frequency (RF)-chain is used to connect all the antenna elements to a transmitter/receiver module in analog beamforming architecture, while in the digital beamforming, a dedicated RF-chain is required for each antenna element. The hybrid beamforming uses a two stage structure, employing both the analog and digital beamforming, thereby enhancing the array gain (analogue beamforming) and mitigating interference (digital beamforming)^4^. All three beamforming types require multi-antenna systems that helps to improve the signal-to-noise-ratio (SNR) when used at a receiver side. Interference can also be mitigated through generating highly directional beams along the desired direction when used at a transmitter side. This work is motivated by advantages offered by the analogue beamforming, either on its own or as a part a hybrid architecture, and here we explored the utilization of Maxwell fisheye lens to propose a unique scalable analogue beamformer solution.

Maxwell fisheye belongs to a class of gradient-index materials, where the refractive index changes with the geometry of the material^5^. Other gradient-index materials including Luneberg Lens^6^, Eaton Lens^7^ and Fresnel lens^8^ utilizes the energy focusing capability of the lens structure at the antenna end, while this work uses energy focusing capability of a Maxwell Fisheye lens at a feed network level. Maxwell fisheye lens is an inhomogeneous optical system, where rays emerging from one point within the lens follow a circular arc-shaped path about the origin (center) of the lens^9^. Maxwell fisheye lens possesses a characteristic that gives impetus to its use in analogue beamforming, i.e., for each point on the Maxwell fisheye lens, there is an image, where the image is a conjugate of the source. The optical length between two conjugate points on the circular arc-shaped trajectory is the same. Therefore, the capability of translating source signal to image signal by the Maxwell fisheye lens motivates its use for developing a self-scalable multi-antenna system feed network that does not require a corporate feed network with a dedicated number of ports to excite spatially distributed antenna elements operating as an array. Specifically, the contributions of this work are as follows: First, we propose a novel beamformer design architecture using Maxwell fisheye lens whose capability of on demand beam shaping and beam steering is demonstrated for the first time; Second, we demonstrate that our proposed approach does not require a fixed corporate feed network and is scalable to handle between 2 to 8 antenna array elements; Third, we validate the beam shaping and beam steering using proof-of-concept prototype. The remainder of the paper is organized as follows. In “Maxwell fisheye lens-based beamformer” section, we discuss the proposed beamformer design, present the operation of the Maxwell fisheye lens and its corresponding theoretical model. Prototype development is explained in “Prototype development method” section, followed by results and discussion in “Results and discussion” section. While “Conclusions and future work” section concludes the paper and presents future directions to this work.

## Maxwell fisheye lens-based beamformer

### Beamformer design

**Figure 1:**
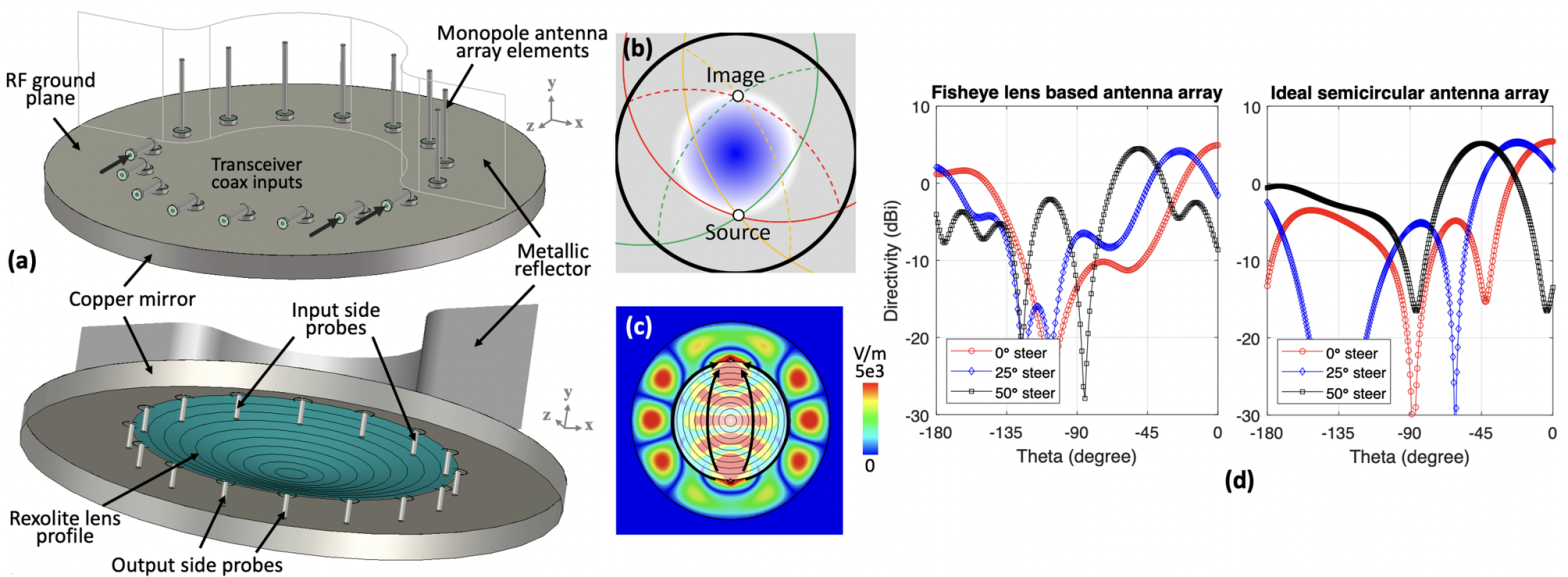
(**a**) Maxwell Fisheye lens based beamformer with transceiver input in first half of the lens, and monopole circular antenna array in the second half of the lens, (**b**) Ray tracing diagram depicting source and image locations in a metallic mirror enclosed Maxwell fisheye lens (**c**) Simulated absolute *E*-field inside the lens structure when a signal at 10 GHz is excited at the source probe of the lens, (**d**) Comparison between far-field directivity of an ideal semicircular antenna array and the proposed Maxwell fisheye lens based beamformer when relative phase shifting given in Table 2 is applied.

The proposed Maxwell fisheye lens based beamforming semi-circular array design is shown in Fig. 1a. The figure shows the transceiver coaxial input emanating from the a metallic sheet acting as a RF ground plane. On the bottom side of this RF ground plane is a planar Maxwell fish-eye lens, where probe of the transceiver coaxial input is exciting the lens. The RF ground plane is bounded by a copper mirror, which is necessary to confine the microwave signal within the lens structure. The circular lens structure can be characterised into two halves, where half of the circle represents the input-side and the remaining half represents the monopole side. The dielectric profile is surrounded by the input and output side probes. At the monopole side half of the Maxwell fisheye lens, coax probes (similar to that of the input-side) are connected to monopole antennas, which are vertically placed at the top side of the RF ground plane. Monopole antennas connected to the output probes of the Maxwell fisheye lens creates a semi-circular monopole antenna array, which is backed by a metallic reflector placed at a distance of $\lambda /2$. The lens structure and monopoles are designed to operate at 10 GHz. Relative locations of the monopole antenna elements are provided in Table 1. The source and drain locations used in this work coincide with the location identification via time reversed scattering transformation approach shown in^10,11^, while general form of geometrical solution of Maxwell fisheye lens can be found in^12^.

### Maxwell fisheye lens operation

To understand the operation of Maxwell fisheye lens and how it is used to develop a beamformer, let us consider a three dimensional Cartesian coordinate system shown in Fig. 1a when the trajectory of the rays within a 3 dimensional (3D) Maxwell fisheye lens is projected onto *xz*- plane^13^. Mathematically, ray trajectory can be represented as $r(t)=x(t), y(t), z(t)$, where *t* signifies the parametric variation of the ray. As shown previously in^14^, the rays from a 3D system projects onto a plane without any loss of generality, and this principle can be used to translate a 3D Maxwell fisheye lens on a finite plane.

The Rexolite lens profile in Fig. 1a is centered at the origin of the Cartesian coordinate system, while the base of the lens as well as the RF ground plane are at $z=0$. By mapping the ray trajectories from spherical points on the *xz*-plane^15^; the differential of the path length on the sphere corresponds to the differential of the optical length on the *xz*-plane. This allowed us to map the geodesic points on multiple circles that produces the sphere to a Maxwell fisheye lens onto the refractive index profile in *xz*-plane, which can be written as^16^

1 $$\begin{aligned} x=\left( \frac{1- sin\theta }{cos\theta }\right) ^2 cos (2\phi ) \text { and } z= \left( \frac{1- sin\theta }{cos\theta }\right) ^2 sin (2\phi ) \end{aligned}$$

**Table 1:** Monopole antenna element position relative to the Maxwell fisheye lens centered at $x=y=z=0$ mm.

Element position	1	2	3	4	5	6	7	8
x (mm)	− 29.4	− 24.9	− 16.7	0	0	16.7	24.9	29.4
z (mm)	0	− 16.7	− 24.9	− 29.4	29.4	24.9	16.7	0

**Table 2:** Phase correction applied to each antenna element.

Element	1	2	3	4	5	6	7	8
Phase ($^\circ$)	− 242.84	− 153	− 53.76	0	0	− 53.76	− 153	− 242.84

In this stereographic projection, the line segments *dx* and *dy* can be represented using spherical coordinates, hence the cylindrical lens profile can be represented as

2 $$\begin{aligned} n^2(x,y,0)[dx^2 + dy^2]=n_0^2[d\theta ^2 + sin^2\theta d\phi ^2] \end{aligned}$$

where, at the equator of the reference sphere index $n = n_0$. Using this principle, the Maxwell fisheye lens profile can be represented by^14,17^:

3 $$\begin{aligned} n=\sqrt{\varepsilon }_r=\frac{2n_o}{1+\frac{r^2}{R^2}}, \quad \mathrm {when} \ r \in [0,\infty )] \end{aligned}$$

where *R* represents the radius of the reference sphere and $2n_o$ is the refractive index at the center of the lens. The traditional methods used to create a refractive index profile for fisheye focusing include thin plates^18^ and holy parallel plates^19^. In this paper, we use mode theory of parallel-plate waveguide^20^ for realizing the fisheye principle. Our design comprises of ideally conducting parallel plates, where the field propagating parallel to the plates has a general form of propagation constant given by

4 $$\begin{aligned} k_m=\sqrt{k^2-m^2\left( \frac{\pi }{d}\right) ^2} \end{aligned}$$

where $k=\omega \sqrt{\varepsilon _r}/c.$ and $m \in {1,2,...}$. The propagation will take place when $k_m$ is real. For $k<\pi \sqrt{\varepsilon _r}/d$, where $k_m$ is imaginary and the propagating waves undergo an exponential decay in the direction of propagation. This leads to development of parallel plate waveguide structure that can act as a high pass filter with a cut-off frequency given by

5 $$\begin{aligned} \omega _c=\frac{m \pi c}{d\sqrt{\varepsilon (\omega )}}, \quad \mathrm {when} \ m \in \{1,2,...\} \end{aligned}$$

where *m* represents the mode number. The wave will propagate at a phase velocity $v=\omega /k_m$ for $\omega >\omega _c$ and energy will be transported at a group velocity $v_g=d\omega /dk_m$. The group velocity can be controlled by creating a graded index profile through Eq. (3). The parallel plate waveguide is partially filled with a dielectric material of permittivity $\varepsilon _r$ with a varying thickness across the lens, which leads to an effective dielectric $\acute{\varepsilon _r}$ given by

6 $$\begin{aligned} \acute{\varepsilon _r}=1-t(r)\left( \frac{1+\varepsilon _r}{d}\right) . \end{aligned}$$

In Eq. (6), *r* denotes the parallel plate’s radius, while *t* is dielectric material thickness. The effective permittivity $\acute{\varepsilon _r}$ can be controlled by the method explained in^21^. Equations (3), (5) and (6) are used to formulate the dielectric profile between the parallel plates shown in Fig. 1a, where coax probes are use to excite a signal at 10 GHz within the parallel plate region.

There is a physical limitation regarding the design of the lens which is related to the near zero index area of the lens profile resulting due to the approach given in Eqs. (3) and (6), which is the requirement of an infinitely large lens to realize the perfect fisheye operation. We mitigate this by including a cylindrical reflecting metal sheet to limit the wave propagation within a finite area as postulated in^22^. Figure 1b demonstrates the function of the mirror where multiple circular ray trajectories from the source are reflected by the mirror and form an image point, while this operation occurs within a confined space. In practical terms, the signal excited at the source point will have an extension at the image as shown in Fig. 1c. It shows that the wave leaving a Maxwell fisheye lens follows a circular path and converges on an image point. This illustrates the focusing capability of the lens, here $85\%$ of the inserted field at the source port can be extracted from the drain probe at operation frequency of 10 GHz. In this case, the electromagnetic field energy loss is related to the imperfect imaging at the drain located at the image point of the lens, which is discussed in^11,23^.

### Beamformer operation

When a 10 GHz signal is simultaneously excited at the transceiver coax inputs, monopole antennas are excited, hence forming a beam along the $-z$-direction. Figure 1d presents the beamforming results validated via full-wave electromagnetic simulation tool CST Microwave Studio, and compared them with that of a mathematical semi-circular antenna array factor. To achieve beam shaping with maximum directivity, phase alignment at the array excitation is required, thus phase shifting depicted in Table 2 is applied at each of the 8 input ports of the beamformer. The results demonstrate that directivity of the Maxwell fisheye lens based antenna array and an ideal circular array with monopoles are comparable, validating the utility of the fish-eye lens structure. Moreover, we also observe that the directivity patterns have significantly high back lobes, prompting a need of metallic reflector shown in Fig. 1a, which is added to redirect the radiated energy towards $-z$-direction. When beam steering is performed in an ideal circular array, the maximum directivity level remains the same, as shown in the Fig. 1d. Although, there is a slight degradation ($<1\hbox {dB}$) in the maximum directivity when the steering angle of $25^\circ$ is applied to the Maxwell fisheye lens based beamformer, that doesn’t effect the beam steering capability. Additionally, it can be observed that the half power beamwidth for an ideal semicircular array stays consistent when beam steering is performed. Finally, for a Maxwell fisheye lens based antenna array we can observe a side lobe at steering angle of $50^\circ$, however with 6.8dB difference from the maximum directivity the overall beamfomring response is comparable with the ideal semicircular antenna array.

## Prototype development method

**Figure 2:**
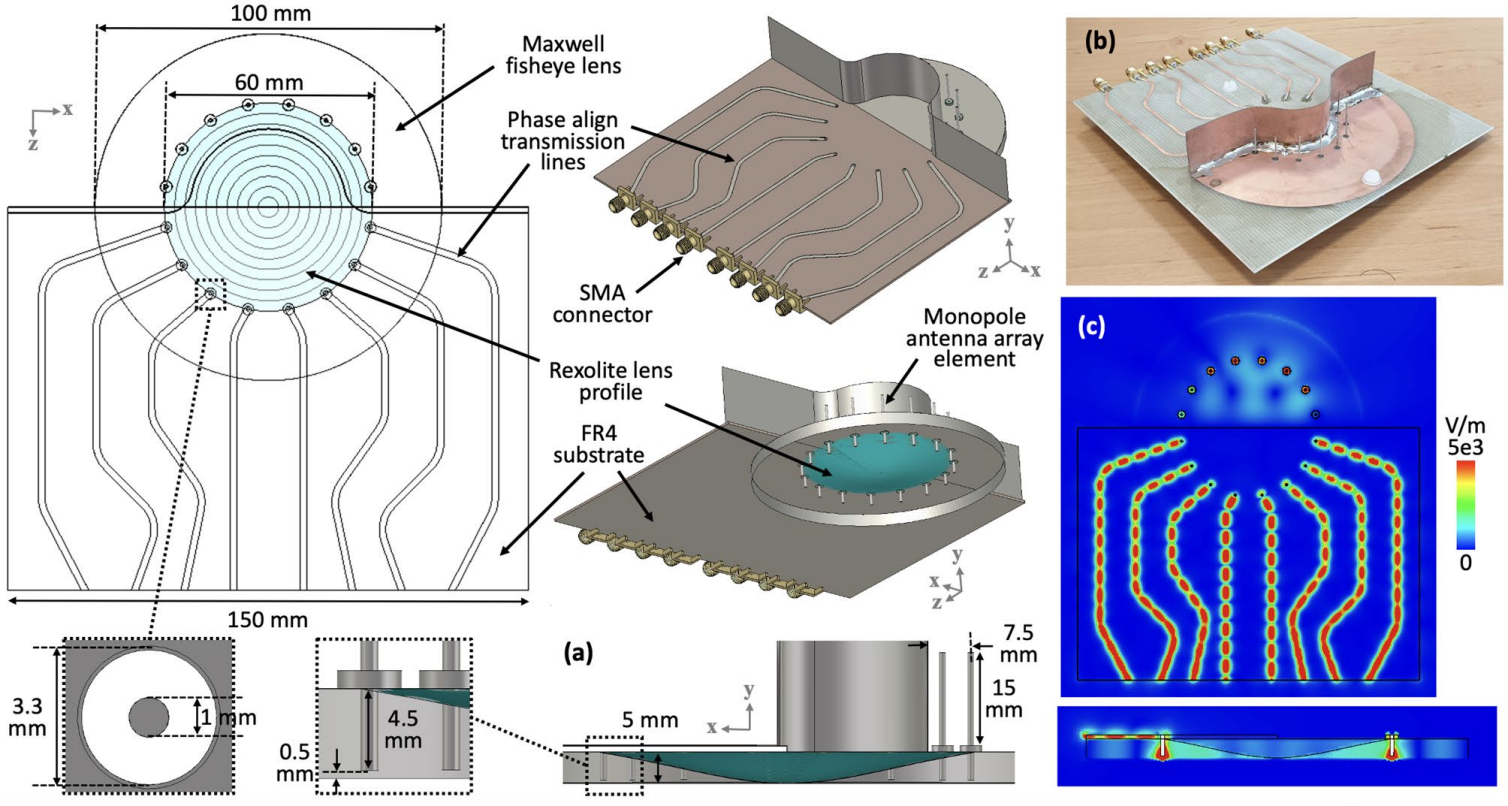
(**a**) Design configuration of the Maxwell fisheye lens beamformer with transmission line network developed for prototyping and performance evaluation, (**b**) Prototype hardware, and (**c**) Simulated *E*-field mapped on multiple 2D surfaces on the beamformer prototype.

Figure 2a shows the dimensions of the Maxwell fisheye lens developed for the beamforming performance evaluation, where the design configuration of the prototype is depicted. The schematic shows the phase aligned transmission lines which are connecting the external signal input through SMA connectors to the inputs of the Maxwell fisheye lens. The monopole antenna array elements are at the output of the Maxwell fisheye lens backed by a metallic reflector when looking at the top side, whereas the Rexolite lens profile is shown when looking at the bottom side of the beamformer in Fig. 2a. Length of the monopole antenna and gap from the metallic reflector is also provided. The Maxwell fisheye lens has two perfect electrically conducting (PEC) plates spaced $d=5$mm apart. Note that the bottom plate from the Maxwell fisheye lens is removed for the purposes of showing the Rexolite lens and probes assembly. The substrate between the parallel plates is Rexolite with $\varepsilon _r = 2.53$, dispersion factor=0.00066 and the coefficient of linear thermal expansion $=3.8 \times 10^{-5} 1/^oF$ inch. These properties make Rexolite a suitable candidate for lens development. A copper mirror surrounds the Rexolite lens, where the diameter of the circular band is 100 mm. The lens is excited through input coaxial probes with a height (*h*) of 4.5mm to provide impedance matching to the 50 $\Omega$ line. Accordingly, the output probes have the same height as the input probes, and the dimension of the input and output coaxial probes are also provided. The lens profile is made from a cylindrical Rexolite stock using Triumph Duplex milling machine. To ensure the best Maxwell fisheye lens performance, we made sure that the surface roughness across the lens profile is $<\lambda /8$ at 10 GHz, while this was realised through polishing the Rexolite lens surface with a Tech-Gen precision finisher. The prototype lens structure has overall diameter of 100mm, whereas Rexolite lens has 60mm diameter. To conduct experiments, we have developed phased aligned module in which all the transmission lines incur the same phase shift. It is important to mention that these phase align transmission lines are used to connect the lens structure to the edge of the board and are not a part of the proposed beamformer. Any length of transmission should work the same way provided that the phase incurred by the propagating wave at 10 GHz is the same.

As in Fig. 1a, in the prototype shown in Fig. 2a, the monopole side of the Maxwell fisheye lens beamformer is where the metal backed monopole antennas are connected to the output of the Maxwell fisheye lens beamformer. Figure 2b show the fabricated prototype used to measure the beamformer performance. Figure 2c shows that the signals travel in the phased align transmission lines independently and the mutual coupling between signals in parallel transmission lines is minimized deliberately. It is important to note that the overall transmission line length is kept as small as possible to avoid any additional signal insertion loss from the edge of the substrate to the input of the Maxwell fisheye lens. Figure 2c also presents a side-view of the transition between the microstrip transmission line, coaxial input, Rexolite lens and coaxial output, where signal propagating through the transmission line is delivered to the input of the monopole antenna through the Maxwell fisheye lens.

## Results and discussion

From the simulations, we observed an impedance mismatch when the reflective mirror is placed close to the probe in the Maxwell fisheye lens. This impacts the device operation and reduces its efficiency. The reason being the classical substrate-filled parallel plate waveguide excitation impedance principle^24^ does not directly apply to parallel plate waveguide with growing/decaying substrate profile, and an additional step of impedance matching optimization is needed. When the probe location and depth is optimized for maximum device efficiency in full-wave electromagnetic simulator, the source excitation forms an image at the drain position. This is done by optimizing close-to-perfect phase alignment of the propagating waves emerging from source side, and terminating at the drain point. In particular, where the Rexolite material is not present, the outer edge of the lens acts purely as a parallel plate waveguide, in which a portion of the signal propagates at uniform velocity.

The solution of wave equation is characterized by *m* in Eq. (5), while the $TE_m$ and $TM_m$ modes are defined on discrete wavelengths, having specific cut-off frequencies. It is pertinent to note that the wave equation solution with no magnetic-fields along the direction of propagation, i.e., $TM_m$ has a special case of having mode $TM_0$ with no cutoff frequency. Hence, the lens structure supports the $TM_0$ mode along the direction of propagation, where this mode doesn’t possess an electric or magnetic field, leading to a quasi-TEM mode. The mode of propagation in the lens structure close to metallic mirror is different from that of within the Rexolite filled parallel plate waveguide (evident from Fig. 1c).

Moreover, the transitions between sections of the lens and other parts of the beamformer prototype contribute differently to the signal propagation, which are: (i) the part of the lens where air transition to the Rexolite substrate; (ii) transition between dielectric filled plates to the coax probe; (iii) transition between microstrip transmission line and source probe, and (iv) the transition between drain probe and monopole antenna array. All of these transitions have different wave propagation characteristics. During prototype development stage, these transitions were separately optimized for maximum power transfer and low return loss operation before combining together in the form of Maxwell fisheye lens-based beamformer shown in Fig. 2a,b.

**Figure 3:**
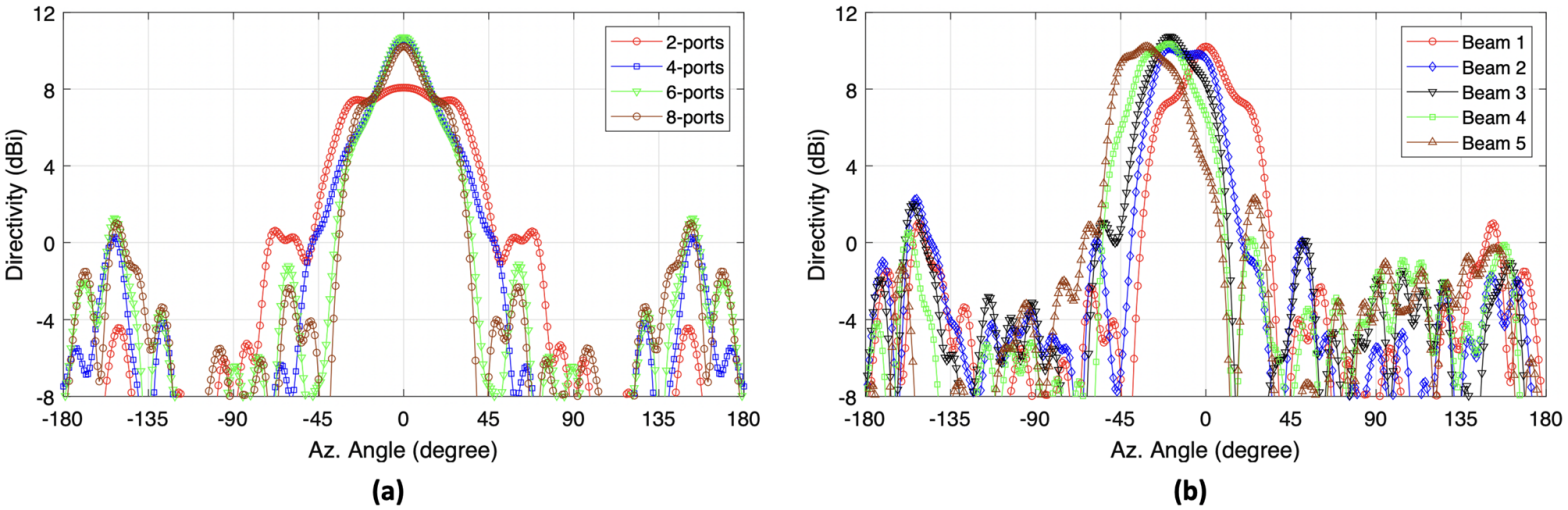
(**a**) Beam shaping achieved when multiple input transceiver ports are excited with same magnitude and phase, (**b**) Beam steering when ideal phase ramp is applied to a shaped beam.

Figure 3 shows the beam shaping and the directivity of the propagating waves when different number of ports of the prototype are excited. The 2 port excitation signifies signal input at two central ports, and so on. It can be observed that the directivity increases as we increase the number of ports from 2 to 8, i.e., a directivity of approximated 6 dBi is observed when 2 ports are excited. The directivity increases to approximately 11 dBi when 8 ports are excited. Moreover, if 2 ports are excited then a similar level of directivity is achieved between $\pm 35^\circ$. When 4 ports are excited, a beam with higher directivity is achievable with a smooth slope between $\pm 50^\circ$. For 6 port excitation, a similar sharp beam shaping is achievable, while the slope can be observed at around $\pm 35^\circ$. It is important to note that the sidelobe level for 4, 6 and 8 port excitation is higher as compared to sidelobe level at 2 port excitation. However, the difference between main lobe and 1st side lobe is around 8 dB. Figure 3a represents the beam steering of Maxwell fisheye lens beamformer,when an ideal phase ramp is applied to all the ports. Although the beam shapes are non-ideal, these are useful for this feasibility study about the behavior of beams steering using this disruptive concept as shown in the Fig. 3b. A phase ramp of $15^\circ$, $30^\circ$, $45^\circ$ and $60^\circ$ is applied separately to form the beam 2, 3, 4, and 5, respectively. It can be observed that when beam 1 is steered towards beam 2 direction, the maximum directivity is slightly reduced from 10.2 dB to 10.1 dB, while the maximum directivity becomes 10.7 dB for beam 3. Furthermore, we observe that beam shifting impacts the beam shape. However, this difference is not significant for this study and the overall device operation is not compromised.

**Figure 4:**
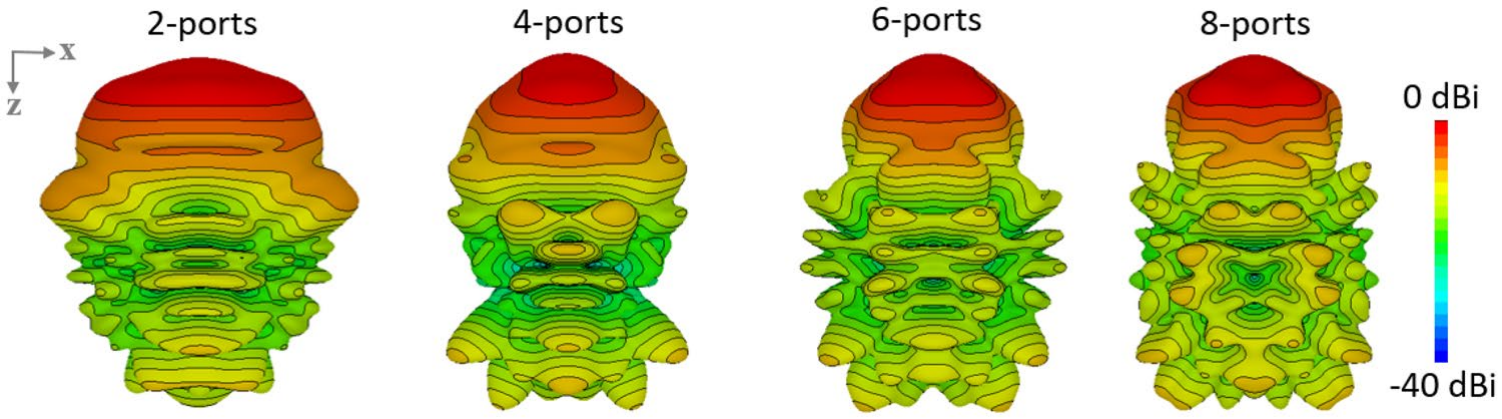
Beam shaping in response to the simultaneous excitation of 2, 4, 6 and 8 ports.

**Figure 5:**
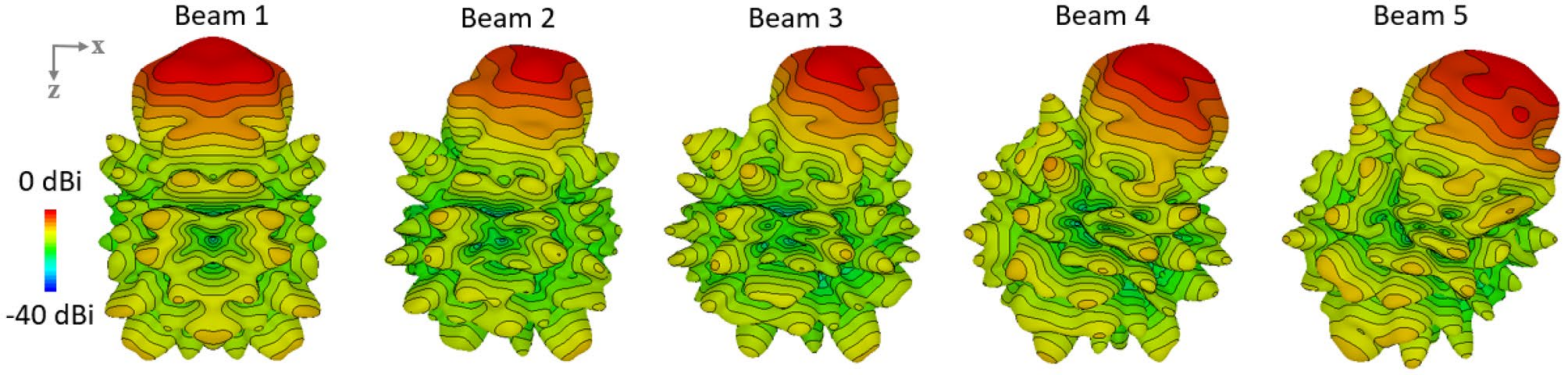
Beam steering as a response to the phase ramp.

Figure 4 depicts the three dimensional directivity view of the beam shaping in response to the excitation of 2,4,6 and 8 ports. The beam becomes narrower as the number of ports excited increases from 2 to 6. While the wide equalized directivity beam can be used to realize broadcast mode, the sharp beam with 6-port excitation can be used for applications demanding high directivity. When all 8 ports of the beamformer were excited, we did not observe any additional benefit for boresight transmission as the beamshaping function is similar to that of the 6 port excitation. Hence it is better to use 6 port excitation to achieve similar operation, while the remaining two ports are interchangeably used for beam steering. The patterns in Figs. 4 and  5 shows contours drawn after every 2dB power difference. The half power beamwidth (HPBW) for the 4 port excitation is narrower when compared to 6 ports excitation, while the maximum area of equal power is wider in 2 port excitation as previously shown in Fig. 3a. In a situation where a narrow beam is required, we can use the 4 port excitation while for a wider beam 2 port excitation can be utilized. The 6 port excitation provides a beam shaping that is intermediary between 2 and 4 port excitation. In all the cases, the sidelobe level is below 8 dB from the main lobe. One important point to note here is that 2, 4 and 6 port excitation can be used for beam steering without using an external phase shifter, whilst the latter is required for beam steering using 8 port excitation and the results are depicted in Fig. 5 which signifies the beam steering in response to the phase ramp applied in a similar fashion as described in Fig. 3a. Beam 1 is generated through an 8 port excitation. The rest of the beams 2,3 and 4 are generated as a result of applying the phase ramp also with 8 ports. It can be observed that the beam steering is achieved but the mean maximum radiation area is disturbed. However, the performance of beam steering is reliable even at higher steering angles, when observed at the 3 dB contour. For example, if we observe beam 5 with higher beam steering, the mean maximum radiation area is still in the desired angular direction i.e. $50^\circ$. This aspect reveals the benefits and flexibility of utilizing the circular array structure.

**Figure 6:**
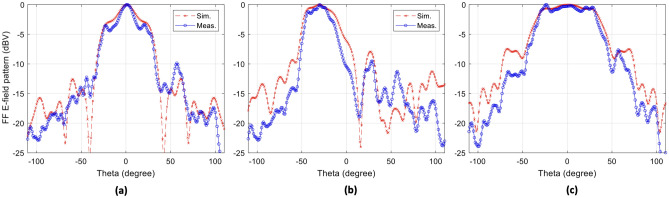
Comparison between simulation and measurement normalized far-field radiation patterns along *yz*-plane.

The prototype shown in Fig. 2b is used to evaluate beam shaping and beam steering capabilities of the Maxwell fisheye lens beamformer. The prototype was placed in a $10\ \mathrm {m}\times 5\ \mathrm {m}\times 5\ \mathrm {m}$ far-field anechoic chamber facility at Queen’s University Belfast, where each port was separately excited by a 10 GHz signal. Co-polarized component in far-field was recorded along the *xz*-plane (same as in Fig. 2a), for all the 8 input SMA ports of the prototype. The far-field measurement data for each port excitation was combined in a post processing step and the normalised results are compared with that of full-wave electromagnetic simulations, shown in Fig. 6. The beam shaping depicted in Fig. 6a,c is close to the simulated predictions. Beam steering depicted in Fig. 6b shows a slight difference in simulated and measured 3 dB beamwidth, while the beam shape and side lobe level is maintained as predicted. The beam shaping for two port excitation resulting in equalized radiations fields along azimuth direction is verified with the help of measurements results shown in Fig. 6c. The measurement results clearly validate the performance of the Maxwell fisheye lens based proposed beamformer hardware.

## Conclusions and future work

In this work, we present a novel scalable analogue beamforming hardware architecture that helps in realizing adaptive beam steering and beam shaping to fulfil the coverage requirements. The proposed beamforming hardware comprises of a finite size Maxwell fisheye lens used as a feed network for a semi-circular array of monopole antennas. The hardware architecture enables on-demand beamforming with a flexibility of using 2 to 8 antenna elements. The unique aspect of the proposed approach is that it does not require dedication of the number of ports in a feed network and is scalable to handle between 2 to 8 antenna array elements. The simulation and measured results are presented to validate the performance of the beamformer in terms of beam shaping and beam steering. As a future direction to this work, we plan to investigate means to achieve better impedance matching between transitions. Moreover, the proposed design can be expanded to act as a high performance wireless repeater due to the simplicity of the feed network.
